# Inorganic Element Determination of Romanian *Populus nigra* L. Buds Extract and In Vitro Antiproliferative and Pro-Apoptotic Evaluation on A549 Human Lung Cancer Cell Line

**DOI:** 10.3390/pharmaceutics13070986

**Published:** 2021-06-29

**Authors:** Brigitta Kis, Ioana Zinuca Pavel, Daniela Haidu, Mariana Nela Ștefănuț, Zorița Diaconeasa, Elena-Alina Moacă, Cristina Adriana Dehelean, Simona Șipos, Alexandra Ivan, Corina Danciu

**Affiliations:** 1 Department of Pharmacognosy, “Victor Babeș” University of Medicine and Pharmacy Timișoara, 300041 Timișoara, Romania; kis.brigitta@umft.ro (B.K.); corina.danciu@umft.ro (C.D.); 2 Research Centre for Pharmaco-Toxicological Evaluation, “Victor Babes” University of Medicine and Pharmacy, 300041 Timisoara, Romania; alina.moaca@umft.ro (E.-A.M.); cadehelean@umft.ro (C.A.D.); 3 Romanian Academy “Coriolan Dragulescu” Institute of Chemistry, 300223 Timisoara, Romania; danielahaidul@gmail.com; 4 Laboratory of Electrochemical and Chemical Technologies, Department of Chemical and Electrochemical Syntheses, National Institute of Research and Development for Electrochemistry and Condensed Matter, 300569 Timişoara, Romania; mariana.stefanut@gmail.com; 5 Department of Food Science and Technology, Faculty of Food Science and Technology, University of Agricultural Science and Veterinary Medicine, 400372 Cluj-Napoca, Romania; zorita.diaconeasa@gmail.com; 6 Department of Toxicology, “Victor Babes” University of Medicine and Pharmacy Timișoara, 300041 Timișoara, Romania; 7 Department of Biochemistry and Pharmacology, Faculty of Medicine, “Victor Babeș” University of Medicine and Pharmacy, 300041 Timișoara, Romania; sipos.simona@umft.ro; 8 Department of Immunology, Faculty of Medicine, “Victor Babeș” University of Medicine and Pharmacy Timișoara, 300041 Timișoara, Romania; ivan.alexandra@umft.ro

**Keywords:** *Populus nigra* L. extract, phenolic compounds, inorganic elements, A549 lung cancer cell migration, apoptosis, cytotoxicity

## Abstract

*Populus nigra* L. is a plant from *Salicaceae* family, native in Europe. Many parts of this tree can be used as active ingredients, but the most valuable are the buds. In recent years, a growing number of studies reported their activity in the development of a wide range of pharmacological activities including diabetes, cardiovascular diseases, and cancer. The aim of this study was to determine the phytochemical composition and to evaluate the inorganic elements’ concentration as well as the in vitro antiproliferative and pro-apoptotic potential of a *Populus nigra* L. buds extract collected from Timișoara (Romania) against A549 human lung cancer cell line. *Populus nigra* L. bud extract was found to contain twelve different phenolic compounds. The inorganic elements concentrations were below the limit of detection for Co, Pb, and As, whereas Cu = 6.66 µg/g; Cr = 0.79 µg/g; Ni = 3.28 µg/g; Fe = 39.00 µg/g; Zn = 14.84 µg/g; Mn = 0.59 µg/g; Al = 2109.87 µg/g; and Cd = 0.019 µg/g. The extract was tested for the in vitro antiproliferative and pro-apoptotic potential on A549 human lung cancer cell line using different concentrations, namely 10, 25, 50, 75, 100, and 150 μg/mL. Results have shown that poplar bud extract induced a significant decrease of tumor cell viability in a dose-dependent manner with an IC_50_ = 72.49 μg/mL and blocked the cells in the G0/G1 phase of the cell cycle. Phenomena of early apoptosis (from 1.34 ± 0.33% control cells to 2.68 ± 0.62% at 150 µg/mL) and late apoptosis (from 1.43 ± 0.14% control cells to 5.15 ± 1.02% at 150 µg/mL) were detected by Annexin V-PI double staining. Poplar bud extract can be regarded as a promising candidate for future studies involving lung cancer.

## 1. Introduction

The long historical use of plants per se or as different types of extracts for their medicinal benefits is well documented in comprehensive reviews on this topic [1,2,3]. To date, there have been a growing number of studies concerning the potential benefits of plant extracts for a wide range of pathologies. Among the most reported biological activities assigned to various plant extracts can be listed the anti-inflammatory, antimicrobial, antidiabetic, cardioprotective, neuroprotective, hepatoprotective, vasorelaxant, hypouricemic, and anti-tumoral properties [4,5].

*Populus nigra* L., commonly known as black poplar, is a well-known medicinal plant, belonging to the *Salicaceae* family. Black poplar is an indigenous tree widespread in almost all continents. In Romania. it grows along rivers, through streams and meadows, and in the regions of hills and plains. In terms of lifespan, black poplar enjoys a true longevity, commonly 300–400 years [6]. Black poplar can be easily confused with white poplar (*Populus alba* L.) and aspen (*Populus tremula* L.). To the extent that during the harvesting period the leaves have not yet appeared, the differentiation will be made after the bark and buds aspect. In the case of the black poplar, the bark forms an early furrowed rhytidome on most of the stem. For the other species, the rhytidome appears only in old trees, the young ones showing a smooth white-gray or gray-green bark. The buds of black poplar are 2–3 cm long and 5–8 mm thick, elongated, and sharp, with a length of 2–3 cm. Due to the resins that cover the surface, they present a shiny appearance and an aromatic smell. For the other species, the buds are ovoid and smaller. The period of harvest is before the development of the leaves, especially from February to April, when the vegetative buds start to swell [7,8,9].

In recent years, a series of studies have been conducted in order to characterize the phytochemical composition of black poplar buds. It has been also underlined the fact that black poplar buds have a number of chemical similarities to propolis (a resinous product collected by bees) [10,11]. Studies on the resin obtained from poplar buds harvested from *Populus nigra* L. have shown the presence of various flavonic derivatives, flavonols, and also flavanones [12,13]. The range of phenolic derivatives identified is also quite wide. Thus, the presence of caffeic, dimethylcaffeic, and isoferulic acids, as well as their esters, was also established. Esters of isoferulic acid with aliphatic and aromatic alcohols are present in significant amounts and appear to predominate in the extract. Among the terpene components identified in poplar buds extract, the most important is bisabolol. In black poplar bark extracts, salicylic acid glycosides, salicortin, salireposide, benzoic acid derivatives, populin (salicin-6-benzoate), and tremulacin (salicin-2-benzoate) were also identified. Additionally, phytochemical investigations have revealed the presence of tannins, triterpene derivatives, wax, α- and β-amirenol, glucose, and fructose [14,15].

Poplar tree species have been extensively studied in different countries because of their beneficial effects on human health. However, the interest in this species has grown mainly in the Western area [16]. Many parts of this tree, such as the buds, leaves, and bark, can be used as active ingredients for the pharmaceutical industry. In European traditional medicine, black poplar buds were generally recommended as a remedy in respiratory infections (cough, laryngitis, bronchitis, sore throat) and also in the treatment of dermatological problems (psoriasis), rheumatic inflammations, and hemorrhoids [17].

In recent years, a growing number of evidence-based studies regarding black poplar bud pharmacological activity have been published. With respect to this direction, this vegetal product was reported to have strong antioxidant activity with a potential similar to the activity of ascorbic acids [18]. The antioxidant activity may be assigned to the high content of phenolic compounds, especially caffeic and isoferulic acids [19,20]. Medicinal plants rich in polyphenols represent a high antioxidant activity due to their hydrogen-donating and metal chelating capacities [21]. It is well known that oxidative stress plays an important role in the development of many diseases including cardiovascular diseases, diabetes, and various types of cancer [22].

Today, a wide range of plant extracts are commonly used. From the point of view of the quality and contained elements, it is also important to verify the content of toxic elements such as As, Cd, Pb, and Hg [23]. The formulation of a phytopharmaceutical product includes several important aspects such as the medicinal plant from which the active principles come (evaluation of the content of toxic elements, especially in the case of plants that are used for phytoremediation) and evaluation of the finished product to define active principles, ensuring that it is not contaminated with toxic metals either from the handling process or from the extractive process [24,25]. The organic composition of black poplar bud extracts has been the focus of some studies, regarding the characterization and quantification of the active compounds present, together with their effects on human health [26,27,28]. In contrast, there are few studies on the characterization of inorganic components of extracts and their potential effects on human health [29]. Recent studies assumed the accumulation of heavy metals in different parts of the plant, such as in roots, where they found more Cd, Cu, and Pb contents than in the leaves or stems. Using black poplar buds as a vegetal product for obtaining a therapeutic extract, we can assume that the risk of contamination is minimal. *Populus nigra* L. presents significant defense mechanisms against Cd, Cu, and Pb with values up to 7.8, 29.8, and 91.1 mg/kg soil. On the other hand, the tolerance index (TI) of either biomass or root was >0.8. Many deciduous plant species are believed to translocate accumulated heavy metals to their above-ground tissues before senescence [30]. However, the quantity of heavy metals that accumulates in the aerial part of the *Populus nigra* L. extract showed appreciable potential for storing heavy metals like Cd and Zn in aging foliage [31,32].

The aim of the study was to conduct a phytochemical characterization of *Populus nigra* L. buds extract (noted further Pg) obtained from the western areas of Romania and to determine the inorganic elements as well as the in vitro evaluation on the A549 human lung cancer cell line in terms of viability, migration, apoptosis, and cytotoxicity.

## 2. Materials and Methods

### 2.1. Plant Materials and Reagents

Pg was collected from Timișoara (Romania) and identified in the Department of Pharmacognosy, University of Medicine and Pharmacy “Victor Babes” (specimen number is Pg 3/2019). The extraction was performed as previously described [33]. Briefly, for the extraction, 10 g of dried Pg was measured and was mixed with 100 mL ethanol 70%. The extract was ultrasonicated in the FALC LBS 2 ultrasonic water bath for 30 min and then filtered with filter paper using a vacuum pump (Vacuubrand). The solvent was evaporated using a rotary evaporator (HEIDOLPH Laborata 4000 efficient WB eco). To earn a dry sample, the Pg extract was placed in an oven for 5–6 h at 50 °C (Genlab N40c).

### 2.2. Phytochemical Composition—HPLC-DAD

ESI-MS (electrospray ionization coupled with mass spectrometry) analysis was performed on an Agilent 1200 system (Chelmsford, MA, USA) that was equipped with a binary pump delivery system LC-20 AT (Prominence), a degasser DGU-20 A3 (Prominence), and a diode array SPD-M20 UV–VIS detector (DAD). The separation of the compounds was achieved on an Eclipse XDB C18 column (4 µm, 4.6 × 150 mm). The mobile phases consisted of solvent A, bidistilled water and 0.1% acetic acid/acetonitrile (99/1) *v*/*v*, while solvent B was acetonitrile and acetic acid 0.1%. The gradient elution system was programmed as follows: 0–2 min, isocratic with 5% (*v*/*v*) eluent B; 2–18 min, linear gradient from 5% to 40% (*v*/*v*) eluent B; 18–20 min, linear gradient from 40% to 90% (*v*/*v*) eluent B; 20–24 min, isocratic on 90% (*v*/*v*) eluent B; 24–25 min, linear gradient from 90% to 5% (*v*/*v*) eluent B; 25–30 min, isocratic on 5% (*v*/*v*) eluent B. Flow rate was set to 0.5 mL/min, and column temperature was maintained at 25 °C. The chromatograms were monitored at 280 and 340 nm. Identification of the compounds and peak assignments were done using their retention time, UV-VIS, and mass spectra, and also when comparing with the commercial standards (chlorogenic acid, caffeic acid, apigenin) and previously published literature. A single quadrupole 6110 mass spectrometer (Agilent Technologies, Chelmsford, MA, USA) equipped with an ESI probe was used for the mass spectrometric measurements. Measurements were performed in the positive mode with an ion spray voltage of 3000 V and a capillary temperature of 350 °C. Data were collected in full scan mode within the range of 100 to 1200 *m*/*z*. For the quantification, the standard curve of chlorogenic acid was used and expressed as a mg/g sample.

### 2.3. Inorganic Element Determination of Pg Extracts by GF-AAS—Sampling and Sample Preparation

Prior to the analyses, all the glassware and plastic vessels were washed properly and rinsed with ultrapure water. About 0.1 g of plant extract was treated with 5.0 mL 67% nitric acid (Sigma Aldrich, Jena, Germany) and subjected to microwave acidic digestion (Table 1). Each determination was performed in triplicate. Metal concentrations were determined using a spectrophotometer novAA 400G (Analytik Jena, Jena, Germany) equipped with a graphite furnace, an auto sampler MPE60, and a Cookbook for all elements. The analyses and data were processed with a WinAAS 3.17.0 soft.

For each element, a calibration curve with standard Merck solutions was previously registered (Table 2). All solutions were prepared with ultrapure water (Barnstead, EASYpure RoDi^®^ apparatus).

### 2.4. Cell Culture

A549 lung adenocarcinoma cell line (ATCC^®^ CCL-185^TM^) was purchased from the American Type Culture Collection (ATCC^®^ PCS-301-010™). The cells were cultured in Dulbecco’s Modified Eagle’s Medium (DMEM; Sigma-Aldrich, (Taufkirchen, Germany) and supplemented with 10% fetal bovine serum (FBS; Sigma-Aldrich, Taufkirchen, Germany) and 1% penicillin/streptomycin mixture (Pen/Strep, 10,000 IU/mL; Sigma-Aldrich, Taufkirchen, Germany) and further maintained in standard conditions (humidified atmosphere with 5% CO_2_ and 37 °C).

### 2.5. MTT Assay

The antiproliferative activity of Pg against A549 cells was assessed using the MTT (3-(4,5-dimethylthiazol-2-yl)-2,5-diphenyltetrazolium bromide) assay. The experiments were effectuated as previously described by Ghitu et al. [34]. Briefly, cells were seeded at a density of 1 × 10^4^ cells/well in 96-well culture plates and allowed to adhere overnight at 37 °C. After 24 h of incubation, A549 cells were stimulated with diverse concentrations of Pg (10, 25, 50, 75, 100, and 150 μg/mL) and incubated for 72 h. Dimethyl sulfoxide (DMSO), the solvent used to make the stock solution, was used as a control in this experiment. At 72 h post-stimulation, the cells were treated with 10 μL of 5 mg/mL MTT solution (Sigma-Aldrich) and incubated for another additional 3 h. The formazan crystals were dissolved in 100 μL of lysis solution, and the absorbance was determined at 570 nm using the microplate reader (BioRad, xMark Microplate Spectrophotometer).

### 2.6. Scratch Assay

To evaluate the regressive effect of Pg on the invasion capacity of A549 lung adenocarcinoma cells, the scratch test was performed [35]. A number of 2 × 10^5^ cells/well were seeded onto 12-well culture plates. When 90% confluence was reached, the attached cells were scratched following the diameter of the well using a sterile pipette tip. Afterward, the wells were washed with phosphate-buffered saline PBS (Thermo Fisher Scientific, Boston, MA, USA), and the cells were stimulated with different concentrations of Pg (10, 25, 50, 75, 100, and 150 μg/mL). In order to compare the cell growth of the stimulated vs. control cells in early stages, the wells were captured on images at 0 and 24 h using the Olympus IX73 inverted microscope provided with DP74 camera (Olympus, Tokyo, Japan). The cellSense Dimension software was used for analyzing the cell migration. The scratch closure rate was calculated using the following formula [34]:

(1) $$\mathrm{Scratch}\;\mathrm{closure}\;\mathrm{rate}=\left[\frac{A_{t_{0}}-A_{t}}{A_{t_{0}}}\right]*100$$

where *A_t_*_0_ is the scratch area at time 0; *A_t_* is the scratch area at 24 h.

### 2.7. Cell Cycle Analysis

In order to distinguish the cell cycle distribution, the cell’s DNA content analysis was determined by FACSCalibur flow cytometer (Becton-Dickinson, Franklin Lakes, NJ, USA). A549 human lung cancer cells were seeded into 6-well plates and were treated with Pg extracts using the different concentrations mentioned above. After 48 h, following trypsinization and centrifugation, cells were collected and fixed with ice-cold 70% ethanol (1000 µL) and stored for 30 min at 5 °C. Prior to analysis, the cells were centrifuged for 5 min at 1500 rpm and re-suspended in PBS. Thereupon, 50 µL of PI (BD Pharmingen, BD Biosciences) was added to the cells and then incubated for 10 min at 4 °C. As a control, cells treated with 0.15% DMSO were used. Using the Flowing software 2.5.1., the percentage of cells present in the different cell cycle (G0, G1, S, and G2) phases was determined [36].

### 2.8. Detection of Apoptosis via 4′,6-Diamidino-2-Phenylindole (DAPI) Staining

The apoptotic potential of Pg at the selected concentrations was analyzed by DAPI staining. A549 lung adenocarcinoma cells were plated onto 6 = well plates (5 × 10^5^ cells/well) and left to adhere overnight. The next day, the medium was removed and a fresh one containing Pg in different concentrations (10, 25, 50, 75, 100, and 150 μg/mL) was added. After 72 h, the cells were washed twice with ice-cold PBS and then were fixed with 4% paraformaldehyde in PBS, permeabilized with 2% Triton- X/PBS for 30 min, and blocked with 30% FCS/0.01% Triton-X. After, as a final step, the cells were washed again with PBS and stained with DAPI (300 nM) in a dark chamber for 15 min. In order to take fluorescent images at a magnification of 40X, we use the fluorescence inverted microscope Olympus IX73, which had an integrated DP74 digital camera (Olympus, Tokyo, Japan) [37].

### 2.9. Annexin V-FITC Apoptosis Assay

Pg extract was examined by flow cytometric analysis with the Annexin V-FITC apoptosis detection kit (Sigma-Aldrich) according to the manufacturer’s protocol. A 6-well plate was used and cells were seeded at the concentrations of 10^4^ cells/well and incubate overnight. The next day, the media were removed and a fresh medium containing the Pg extracts (10, 25, 50, 75, 100, and 150 μg/mL) was added. After 72 h, the cells were trypsinized, washed twice with BB (Binding Buffer), centrifuged at 1500 rpm for 7 min, and resuspended in BB and then incubated with 5 µL of Annexin V-FITC for 15 min at room temperature. After the washing steps, the pellet was resuspended in a 190 μL BB, and immediately 10 μL PI (propidium iodide) solution was added prior to analysis by flow cytometry. The solvent DMSO (0.15%) was used as the control. A549 cells were examined by flow cytometry (FACSCalibur, Becton Dickinson) using the fluorescence channel FL1 for Annexin and FL2 for PI, and results were interpreted using Flowing Software 2.5.1. [38].

### 2.10. LDH Assay

The LDH (lactate dehydrogenase) assay (11644793001 Roche) was performed to determine the cytotoxic effect of Pg using different concentrations (10, 25, 50, 75, 100, and 150 μg/mL). The experiment was performed according to the manufacturer’s protocol. For this experiment, a number of 5 × 10^3^ cells/well were seeded in 96-well culture plates and left to adhere overnight. On the next day, the cells were stimulated with the above-mentioned concentrations of Pg and incubated for 72 h. The next step consists of transferring 100 μL from each well into a new 96-well culture plate, mixing with 100 μL/well of the reaction mixture, and further incubating for 30 min at room temperature. Using a microplate reader (BioRad, xMark Microplate Spectrophotometer), the level of LDH release in the medium was measured at 490 and 680 nm. To establish spontaneous and maximum release of LDH, cells treated with 1% (*v*/*v*) Triton X-100 (high control) and untreated cells (low control) were used [34].

### 2.11. Statistical Analysis

The data obtained in the present study are expressed as mean ± standard deviation. A one-way ANOVA test followed by Dunnett’s multiple comparison test was used for comparison among groups. GraphPad Prism 5 (GraphPad Software, San Diego, CA, USA) was used for the statistical analysis.

## 3. Results

### 3.1. Phytochemical Composition

**Figure 1:**
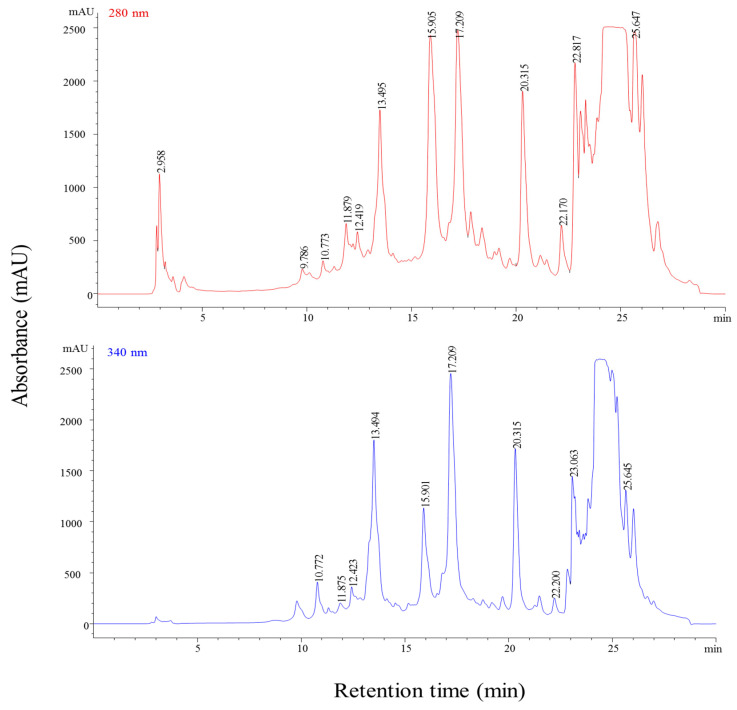
LC chromatogram of Pg extract (20 mg at 280 and 340 nm).

The extract showed a high content in phenolic compounds. Liquid chromatography (LC) separations of the phenolic compound are presented in Figure 1 and their identification in Table 3. The Pg extract was found to contain twelve different phenolic compounds consisting of dihydroxybenzoic acid, protocatechuic acid, 3-caffeoylquinic acid, 5-caffeoylquinic acid, caffeic acid, chicoric acid, apigenin-glucuronide, chrysoeriol-glucuronide, tremuloidin, salicin, pinostrobin, and tremulacin. The identification was further confirmed by comparison of their chromatographic retention times and mass spectrometry (MS) fragmentation spectra with those of standards and data available in the literature [39,40]. Prior to LC separation, Peak 1 (Rt = 2.96) with an [M − H]^+^ ion peak at *m*/*z* 155 in the ESI mass spectrum was confirmed as a dihydroxybenzoic acid. Further, the mass spectral value of compound **2** showed a quasi-molecular ion [M − H]^−^ at *m*/*z* 155 with a UV max at 280 nm that was identified as a protocatechuic acid. The electrospray ionization coupled with mass spectrometry (ESI-MS) of compounds **3** and **4** showed a quasi-molecular ion [M − H]^−^ at *m*/*z* 355; both are the same mass value, only the position is different; those compounds were identified based on available standards as 3-caffeoylquinic acid (neochlorogenic acid) and 5-caffeoylquinic acid (chlorogenic acid), respectively.

Next, two more hydroxycinnamic acids were identified (compounds **5** and **6**) corresponding to caffeic and chicoric acid, confirmed with MS via the ion *m*/*z*, while the next three peaks (**7**, **8**, and **9**) were flavone derivatives that defined a fragment at *m*/*z* 447, 477, and 285, respectively, corresponding to apigenin–glucuronide, chrysoeriol–glucuronide, and tremuloidin. Compound **10** was detected as salicin, while compound **11** was pinostrobin, which belongs to the flavanone class. The last compound with ultraviolet-visible (UV-VIS) data (λ max 312, 240) showed an [M − H]^+^ ion at m/z 271; this was identified as tremulacin. Quantitative analysis revealed that the analysis sample was very high in flavone, as depicted in Table 4. Apigenin–glucuronide was demonstrated to be a major compound, found in an amount of 55.828 mg/g chlorogenic acid equivalent (CCE). Chry-glucuronide and tremuloidin were also the most abundant compounds among all the phenolics identified.

### 3.2. Inorganic Elements

Results show that in the tested sample, the concentrations of the obtained elements vary from 0.019 µg/g for Cd to 2109.87 µg/g for Al (Table 5). The used measurement unit is µg/g (which is the equivalent of ppm and mg/kg). Arsenic, lead, and cobalt were below the limit of detection.

### 3.3. MTT Assay

**Figure 2:**
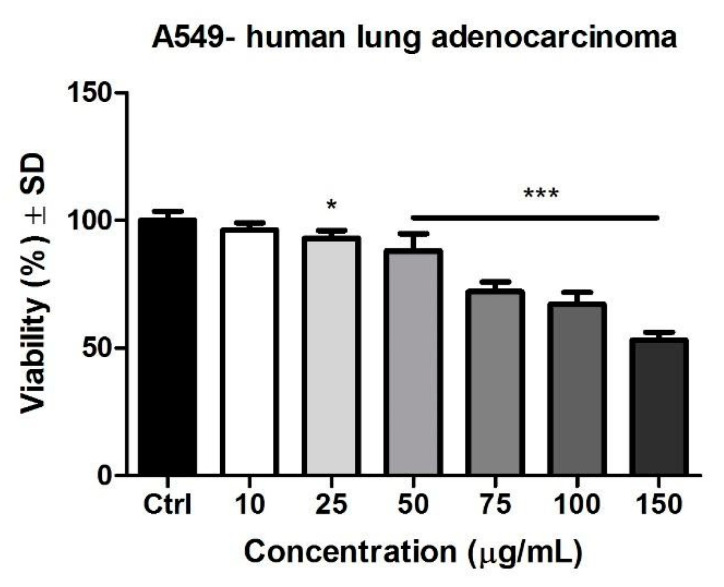
Pg (10, 25, 50, 75, 100, and 150 μg/mL) effect on A549 human lung adenocarcinoma cells viability after 72 h stimulation. The data are expressed as cell viability percentage (%) related to the control cells. A one-way ANOVA test and Dunnett’s multiple comparison post-test were employed for comparison among groups (* *p* < 0.05, *** *p* < 0.001 vs. Control).

The effect of Pg was evaluated on the A549 lung adenocarcinoma cell line following a stimulation period of 72 h with the selected concentrations. Results obtained on the tumor cell line are depicted in Figure 2. It can be observed that Pg provoked a dose-dependent decrease of tumor cells’ viability, the highest decrease of viability being 53 ± 3.1% (at 150 μg/mL) vs. control. A significant decrease of tumor cell viability was also obtained at 25 μg/mL (93 ± 2.9%), 50 μg/mL (88 ± 6.7%), 75 μg/mL (72 ± 3.8%), and 100 μg/mL (67.2 ± 4.5%). The IC_50_ value calculated using GraphPad Prism was 72.49 μg/mL (Table 6).

### 3.4. Scratch Assay

**Figure 3:**
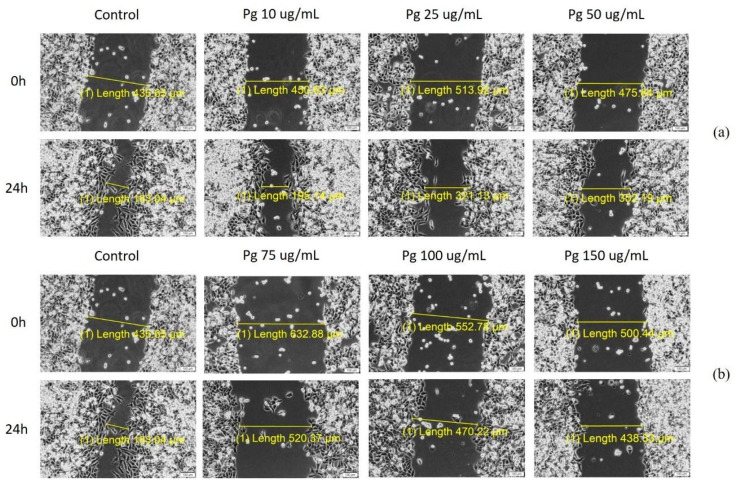
Pg extract (**a**) (10, 25 and 50) and (**b**) (75, 100, and 150 μg/mL) activity on A549 human lung adenocarcinoma cells’ migration and proliferation potential. Progression of cell migration was monitored by imaging the scratch line initially and at 24 h post-stimulation. Images were taken by light microscopy at 10× magnification.

The potential anti-migratory effect of Pg extract on A549 lung adenocarcinoma cells was determined by means of a scratch assay. Results have shown that this extract decreased tumor cell migration in a dose-dependent way, and at the highest dose tested (150 μg/mL), it produced changes in A549 cells’ morphology (Figure 3).

**Figure 4:**
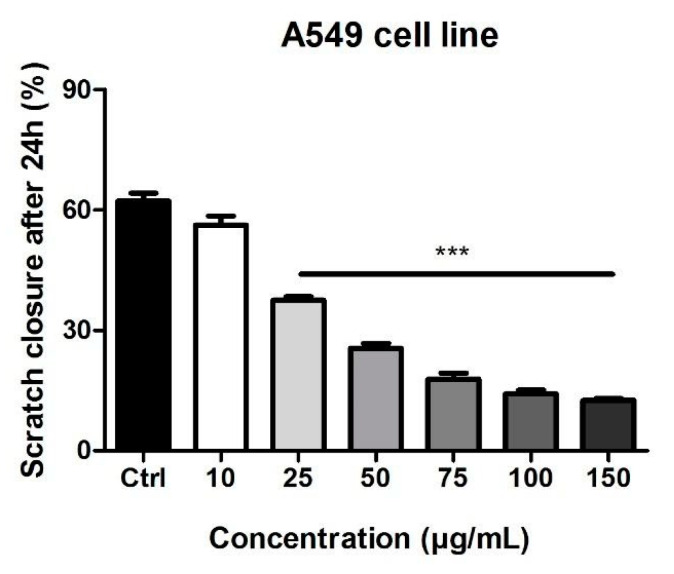
The anti-migratory potential of Pg extract (10, 25, 50, 75, 100, and 150 μg/mL) on A549 lung adenocarcinoma cells. The bar graphs are expressed as percentage of scratch closure after 24 h compared to the initial surface. One-way ANOVA test and Dunnett’s multiple comparison post-test were employed for comparison among groups (*** *p* < 0.001 vs. Control).

Figure 4 represents the scratch closure rate for the selected concentrations. It can be noticed that Pg produced a decrease in the closure rate in a dose-dependent manner, indicating that the extract elicited an anti-migratory effect on A549 lung adenocarcinoma cells. At the highest doses, the scratch closure rate was 14.1% for 100 µg/mL and 12.5% for 150 µg/mL.

### 3.5. Cell Cycle Analysis

**Figure 5:**
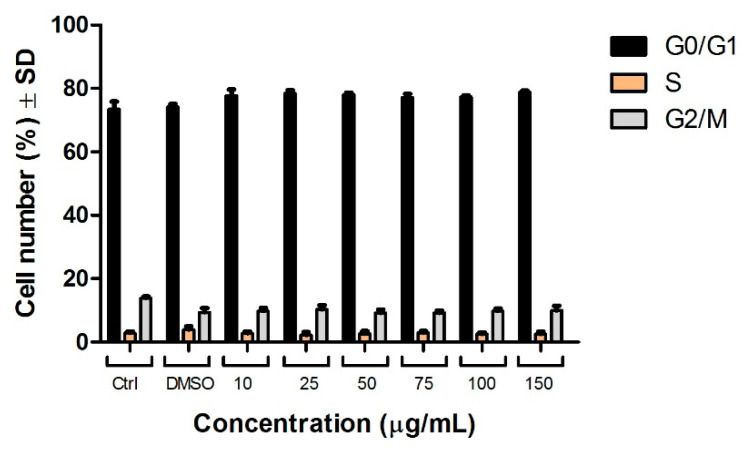
A549 human lung adenocarcinoma cell cycle analysis after stimulation with Pg extract (10, 25, 50, 75, 100, and 150 μg/mL) on G0/G1; S and G2/M phases. The results are expressed as cell number percentage (%) ± standard deviation.

**Figure 6:**
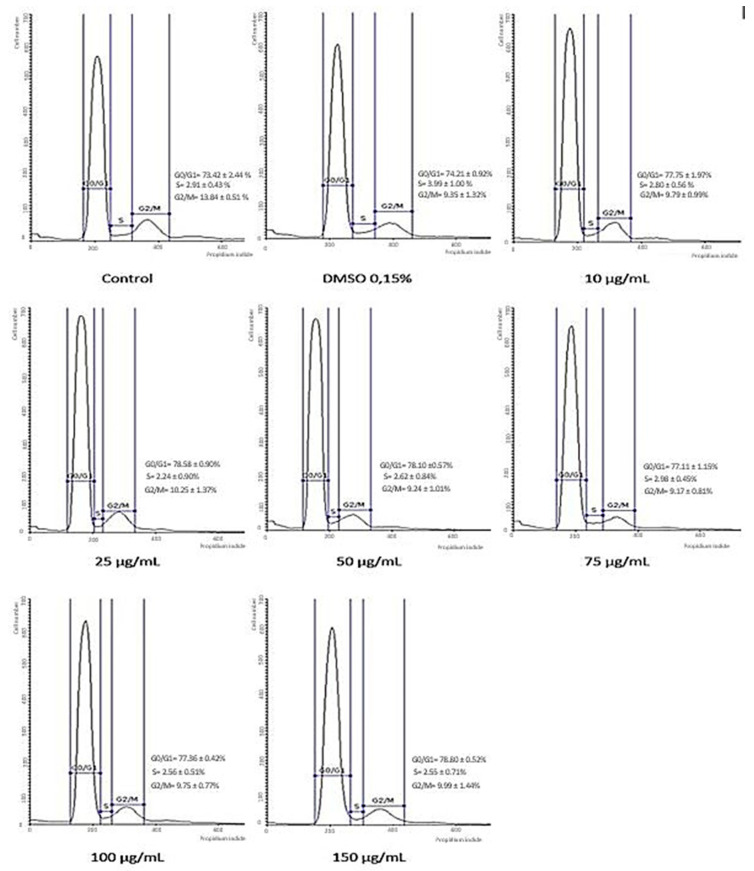
Cell cycle histograms determined by flow cytometry. Effects of Pg extract (10, 25, 50, 75, 100, and 150 μg/mL) on cell cycle phases (G0/G1, S, and G2/M) after 72 h exposure to Pg extract for A549 human lung adenocarcinoma cell line. Results are expressed as cell viability percentage (%) related to the control cells.

In order to understand the effect of the ethanolic extract of Pg on the A549 cell cycle profile, the nuclear DNA content of the cells was analyzed by flow cytometry following 72 h treatment. DMSO was used as a solvent control for the assay, while untreated cells were used as a negative control. A tendency to block the A549 human lung cancer cells in the G0/G1 phase of the cell cycle could be observed (Figure 5). The percentage of cells in this phase was slightly increased from 73.42% ± 2.44 (control cells) to 78.80 ± 0.52 (150 µg/mL Pg) after treatment with the Pg extract. Simultaneously, the percentage of cells in the mitosis phase (G2/M) decreased from 13.84% ± 0.51 in the control cells to 9.99% ± 1.44 for the cells treated with the highest tested concentration (150 µg/mL Pg) of Pg. Figure 6 presents the histogram of cell cycle analysis. Results are presented as the mean of three independent experiments ± standard deviation.

### 3.6. DAPI Staining

**Figure 7:**
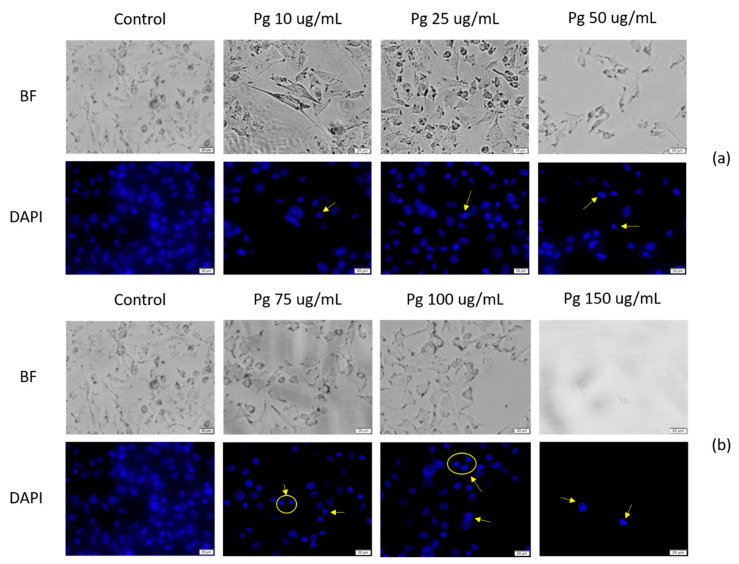
A549 human lung adenocarcinoma cells treated with Pg extract at different concentrations (**a**) (10, 25 and 50) and (**b**) (75, 100, and 150 μg/mL), for 72 h-DAPI staining was performed for apoptotic morphological characteristics; BF = bright field microscopy. The scale bars represent 20 μm.

The taken images (Figure 7) indicate that there is chromatin condensation following stimulation with Pg extract. The control showed a normal organization and a uniform chromatin density. Under the current experimental conditions, deoxyribonucleic acid (DNA) fragmentation was not observed. The data obtained show that after treatment with Pg extract, the A549 tumor cells manifested signs of apoptosis.

### 3.7. Annexin PI

**Figure 8:**
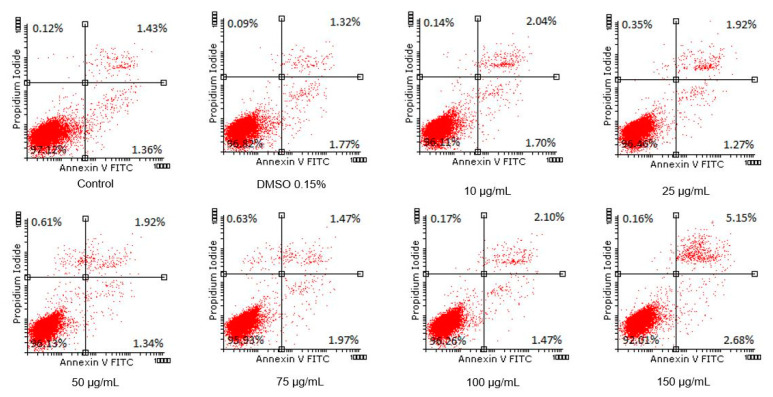
Representative dot-plots. Viability of A549 human lung adenocarcinoma cell line using Annexin V/PI analysis.

**Figure 9:**
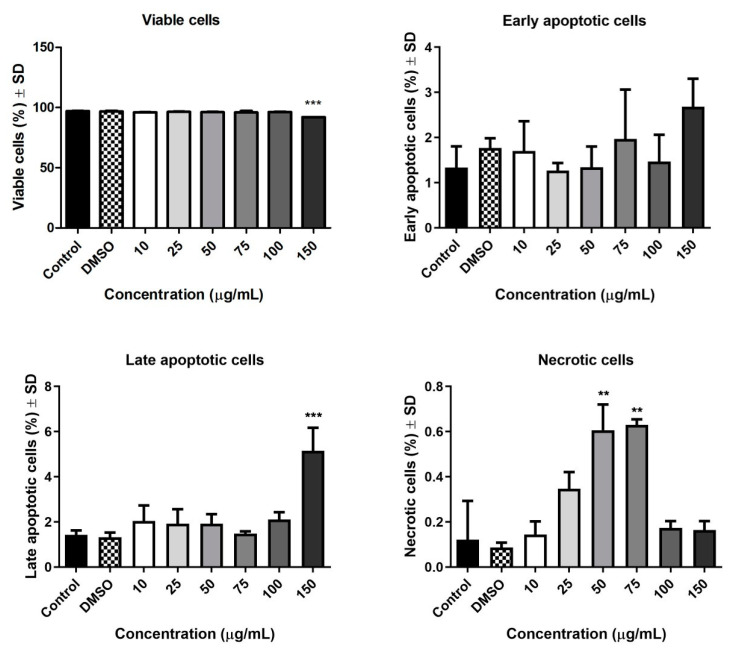
The pro-apoptotic potential of Pg extract (10, 25, 50, 75, 100, and 150 μg/mL) on A549 human lung adenocarcinoma cells. The bar graphs are expressed as the percentage (%) of viable cells, early apoptotic cells, late apoptotic cells, and necrotic cells related to control cells. ** *p* < 0.01, *** *p* < 0.001.

In order to verify whether the Pg extract has an effect on early apoptosis, late apoptosis, or necrosis, the Annexin V-FITC/PI assay was performed using flow cytometry. Figure 8 presents the flow-cytometry dot-plots for the cells treated with different concentrations of Pg (10, 25, 50, 75, 100, and 150 µg/mL), control, and DMSO. A very slight effect of apoptosis induction was observed after cell exposure to the Pg extract; the percentage in early apoptotic cells after 72 h treatment with Pg extracts increased from 1.34 ± 0.33% (untreated cells) to 2.68 ± 0.62% (cells treated with the highest concentration). The percentage of early apoptosis cells after Pg extract treatment are control—1.34 ± 0.33%, DMSO—1.77 ± 0.15%, 10 µg/mL—1.70 ± 0.47%, 25 µg/mL—1.27 ± 0.12%, 50 µg/mL—1.34 ± 0.33%, 75 µg/mL—1.97 ± 0.77%, 100 µg/mL—1.47 ± 0.42%, and 150 µg/mL—2.68 ± 0.62%. Regarding the percentage of late apoptotic cells, a slight increase from 1.43% ± 0.14 (control cells) to 5.15% ± 1.02 (the highest concentration of 150 µg/mL) was observed, while the other concentrations tested did not produce a notable increase in late apoptotic cells’ percentage. The percentage of late apoptosis cells are as follows: control—1.43 ± 0.14%, DMSO—1.32 ± 0.15%, 10 µg/mL—2.04 ± 0.49%, 25 µg/mL—1.92 ± 0.45%, 50 µg/mL—1.92 ± 0.30%, 75 µg/mL—1.47 ± 0.08%, 100 µg/mL—2.10 ± 0.24%, and 150 µg/mL—5.15 ± 1.02% (*p* < 0.001) (Figure 9). The percentage of viable cells decreased from 97.12 ± 0.04% in the case of untreated cells (control) to 92.01 ± 0.02 % in the case of exposure to 150 µg/mL of Pg extract, while the percentage of necrotic cells was significantly increased after treatment with Pg extract at the concentrations of 50 and 75 μg/mL (control—0.12 ± 0.12%, 50 µg/mL—0.60 ± 0.11%, and 75 µg/mL—0.62 ± 0.02%,). Correlated to the above-mentioned determinations, the study proved that, statistically, Pg extract at the highest concentration tested (150 µg/mL) can induce phenomena of late apoptosis when compared to control cells.

### 3.8. Determination of the Cytotoxic Potential by LDH Release

**Figure 10:**
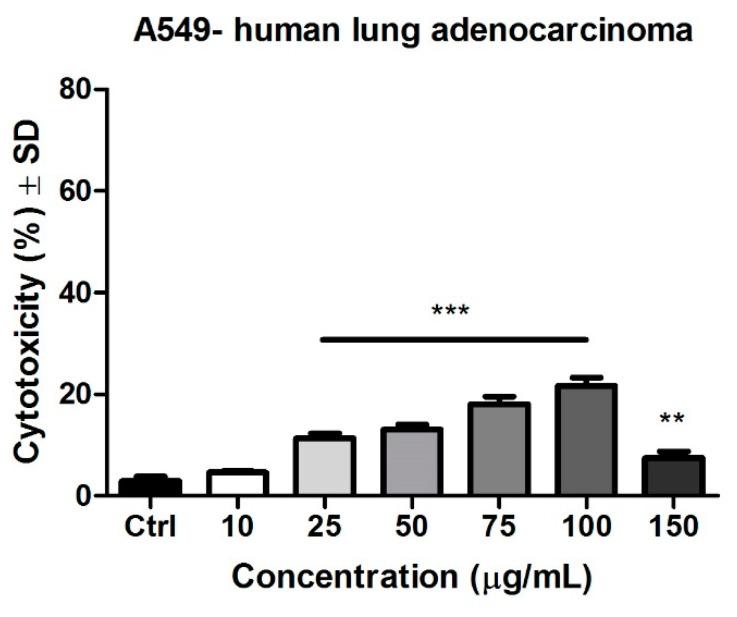
Pg (10, 25, 50, 75, 100, and 150 μg/mL) cytotoxic effect on A549 human lung adenocarcinoma cells after 72 h stimulation. The data are expressed as cytotoxicity percentage (%) related to the control cells. One-way ANOVA test and Dunnett’s multiple comparison post-test were employed for comparison among groups (** *p* < 0.01, *** *p* < 0.001 vs. Control).

To assess the cytotoxic potential of Pg extract, the lactate dehydrogenase assay was conducted. After 72 h of stimulations, the cytotoxicity assessment indicated that Pg extract increased in a dose-dependent manner the release of lactate dehydrogenase. Figure 10 depicts the effect of Pg on the lung tumor cells. A significant cytotoxic effect was obtained at 25 μg/mL (11.3 ± 0.9%), 50 μg/mL (13.1 ± 1%), 75 μg/mL (18 ± 1.4%), and 100 μg/mL (21.7 ± 1.6%). At the highest tested dose (150 μg/mL), the cytotoxic rate was 7.8 ± 1.3% vs. control (2.9 ± 0.8%).

## 4. Discussion

The *Salicaceae* family includes the *Populus* and *Salix* genera, which are widely distributed in Northern temperate regions. Tawfeek et al. reported that the *Salicaceae* family contains different phytoconstituents such as flavonoids, phenolic glycosides (salicin, tremuloidin, and tremulacin), phenolic acids (*p*-hydroxybenzoic, salicylic, gentisic, vanillic, p-anisic, gallic, and protocatechuic acids, but also p-coumaric, isoferuolic, feruolic, and caffeic acids), anthocyanins, procyanidins, organic acids, fatty acids, and volatile compounds (terpenes and monoterpenes) [41]. The literature reported that *Caesaria arborea* L. leaves’ methanolic extract (*Salicaceae* family) contains diterpene, flavonoids, phenolics, and glycoside derivatives (flavonoid-3-*O*-glycosides) [42]. A recent study highlights that the two genera from the *Salicaceae* family (*Populus* and *Salix*) contain mostly secondary compounds. In this regard, the authors characterized the chemical composition of leaves and fruits from two different species, namely *Salix acmophylla* L. and *Populus euphratica* L. Using GS-MS, the group have shown that the phytochemicals in both species varied between phenols (salicin, coumarin, *p*-xylene, and styrene), fatty acids (linolenic acid, 1-octacosanol, and oleic acid amide), amino acids (thymine, isobutyl nitrite, and sebacic acid), alkanes, alkaloids, esters, and carbohydrates (sucrose, hexose) [43]. Djouossi et al. showed that the methanol extract of the leaves of *Oncoba spinosa* Forssk. (*Salicaceae* family) includes flavonoids such as kaempferol, apigenin-7-*O*-β-d-glucuronopyranoside, quercetin, quercetin 3-*O*-β-d-galactopyranoside, and also quercetin 3-*O*-α-l-rhamnopyranosyl [44]. Pg extract evaluated in this study had a high number of phenolic compounds (chicoric acid, apigenin-glucuronide, chrysoeriol-glucuronide, tremuloidin, dihydroxybenzoic acid, protocatechuic acid, 3-caffeoylquinic acid, 5-caffeoylquinic acid, caffeic acid, salicin, pinostrobin, and tremulacin). Results obtained in this study regarding the phytochemical composition of the ethanolic extract of Romanian poplar buds are in line with those reported by other authors.

Poplar is known to be an efficient bio-accumulator of soil chemicals, allowing its use to remedy the soil of toxic elements; because of this property, it was the subject of many studies [45,46,47]. However, its ability to bioaccumulate raises concerns about the possible adverse health effects of products derived from this plant that are ingested by humans, in various forms, used for medicinal purposes. The active principles are the ones generally sought and highlighted, but from the point of view of the contained elements, it is important to determine the concentration of trace elements (Mg, Fe, Mn, Cu, and Zn) and potentially toxic elements (Al, Co, Cr, As, Cd, and Pb). Results obtained for the screened extract are compared with the maximum allowed doses for herbal supplements, but also with the recommended values for drinking water (for the elements where there are no recommendations for herbal medicines). Assuming that the daily water intake is 2 L, in this way, we can estimate a maximum allowable concentration per day, and we can compare it with the concentration obtained in the alcoholic extract of poplar buds. Additionally, for trace elements, the recommended daily dose is used comparatively. There are several regulations stipulated by WHO guidelines for assessing the quality of herbal medicines with reference to the contaminants such as As, Cd, and Pb. The National Sanitation Foundation recommends that a finished dietary supplement (such as poplar buds ethanolic extract) may bring a maximum intake of 0.01 mg/day of As, 0.006 mg/day of Cd, 0.02 mg/day of Pb, and 0.02 mg/day of Cr [48]. The first important step for a phytopharmaceutical product is the validation of being free of toxic metals. As is a toxic metal, and chronic exposure is associated with neuropathy, developmental disabilities, decreased IQ, numerous skin disorders, hypertension, and cancer of the skin, lungs, bladder, and kidney [49]. In the extracts, it has a concentration below the detection limit, namely below 13.2 µg/L. Moreover, knowing this, the As concentration from the Pg ethanolic extract does not represent a reason for concern and does not contribute to the accumulation process. Lead (Pb) is another toxic metal. Enhanced Pb concentration in blood affects cognitive performance, behavior, and postnatal growth and reduces hearing capacity in children and infants. In adults, Pb causes kidney, cardiovascular, fertility, and central nervous system disorders. WHO accepted for an adult a daily Pb intake of up to 7 µg/kg body weight or 490 µg of Pb [50]. In Pg ethanolic extracts, Pb concentration was below the detection limit set at 7.4 µg/L. Knowing the WHO recommendations [48] of a maximum of 10 ppm for herbal supplements, it can be assessed that the ethanolic extract can be used safely.

It is known that for nonsmokers, diet is the major source of cadmium (Cd), which is a food-chain contaminant. Chronic Cd exposure is usually associated with chronic kidney disease, cardiovascular disease, diabetes, osteoporosis, and cancer [51]. Cd principally accumulates in the kidneys, but blood Cd levels show a correlation with urinary Cd levels, and they could thus be of value in risk assessment [52]. Cd was detected in a concentration of 0.019 µg/g, the equivalent of 0.019 ppm, less than the permitted limits by WHO [46] of 0.3 ppm for herbal supplements. Cobalt is an important oligoelement, widely dispersed in the environment in low concentrations. Most people are exposed to small amounts of cobalt by consuming contaminated drinking water or food or inhaling contaminated air. The only known biological function of cobalt is its role as a metal component of vitamin B12, also named cyanocobalamin [53]. Exposure of humans and animals to levels of cobalt normally found in the environment is not harmful. It is known that water quality standards for cobalt (Co) have not been developed for the European Union or United States [54]. From this point of view, Co concentration in poplar buds’ ethanolic extract was under the detection limit (5.4 µg/L), being safe to use as a therapeutic remedy.

*Primula officinalis* L. flower ethanolic extract presented a similar copper (Cu) concentration (6.9 mg/kg) [29] as Pg extract from the present study: 6.66 µg/g. Usually, there is less Cu intake, so any Cu supplementation in food is good. Accumulation of nickel in the body through chronic exposure from highly nickel-polluted environments may be responsible for cancer of the respiratory tract, lung fibrosis, cardiovascular, and kidney diseases. Natural nickel deficiency does not easily occur, as it is found in abundance in food [55]. In Pg ethanolic extract, the nickel concentration was found to be 3.28 µg/g, a higher concentration compared to the summarized concentrations by Pohl et al. [56] for herbal tea infusions or decoctions: 0.0003–0.42 µg/mL, but still low compared to WHO recommendations [57] for drinking water: 20 µg/L Ni. Aluminum (Al) is present in processed and unprocessed foods and drinks, but its low absorption from the intestinal tract and removal in urine via the kidneys make the levels of Al present in the body remain low, without concern for people with normal kidney function. Al can cross the blood–brain barrier, so it can accumulate in the brain. For example, neurotoxic effects have been observed in animal experiments at doses above 200 mg/kg bw/day; therefore, based on the available data, the European Food Safety Authority (EFSA) carried out a safety evaluation and did not consider exposure to Al to constitute a risk for developing Alzheimer’s disease. Additionally, the tolerable weekly intake (TWI) of 1 mg/kg body weight (bw)/week was increased, because of their confidence in the risk assessment to 2 mg/kg bw/week [58]. In Pg ethanolic extract, aluminum reaches a concentration of 2109.87 µg/g, equivalent to 2.1 mg/g. This is not alarming, because it is reported that in the case of drug administration, the normal average intake may reach 50–1000 mg/day [59]. Additionally, some in vitro methods suggest that Al from a tea infusion is potentially available for absorption at only 4.8%. Moreover, only 0.37% is orally bioavailable, established by in vivo experiments [60]. Chromium is a potentially toxic element, but keeping it under a safe limit is a major challenge because of the metal bioaccumulation in medicinal plants, consequently leading to human health exposure. Nirola et al. [61] mention that the acceptable Cr concentration for raw medicinal plants used further for their therapeutic values is up to 10 µg/g. A manganese (Mn) concentration of 0.59 µg/g and a chromium (Cr) concentration of 0.79 µg/g do not have a large contribution to the dietary intake, because the recommended dietary allowances (RDA) are 2.30 mg Mn/day/person and 35 µg Cr/day/person for males [62]. Even the WHO recommendations [56] for drinking water provide a maximum of 50 µg/L Mn, thus being higher than the one obtained for the plant extract in this study. Iron (Fe), an important trace element, is a vital nutrient for the human body. It takes part in a variety of cellular events and activates many enzymes responsible for the synthesis of collagen, amino acids, neurotransmitters, and hormones. The iron content in the body must be equilibrated because any disequilibrium like excess or deficit may have harmful consequences. The poplar buds’ ethanolic extract contains 39.00 µg/g. This value is small, and considering that Fe is classified among the poorly extractable elements (below 20%) and that the TDI for an adult is 0.7 mg/kg body weight (49 mg for an adult) [60], the Fe intake from the extract is negligible. This conclusion is certificated by the comparison with the contents of a survey of plant iron content, mentioned for plants like *Tectona grandis* L.f., *Amaranthus spinosus* L., and *Stylosanthes erecta* P. Beauv., which contained the highest iron contents: 2666, 2366, and 2066 µg/g, respectively [63]. Zinc (Zn) is a trace metal, one of the metals that accumulates even in high concentrations in the plant, in the phytoremediation process. In this manner, Guerra et al. [64] reported that for *P. nigra clones*, zinc (Zn) leaf concentrations ranged within the interval 569–935 µg/g. In the present study, the detected Zn concentration (14.84 µg/g) represents a low intake of Zn, but at the same time, it is risk-free if up to 88.8% can be extracted from the plant by ethanolic extraction [29].

Thus, based on experimental outcomes, it can be concluded that the Pg ethanolic extract has a low contribution to trace elements in dietary intake, but the most valuable conclusion is regarding its safety; we can highlight that Pg extract does not produce any harmful effect of metal toxicity during its therapeutic application.

As is already known, cancer is one of the most frequent diseases caused by genetic alterations in cells [65]. Lung cancer is one of the most common causes of death in both men and women worldwide [66]. Many natural compounds have been shown to be effective in the management of various types of cancer, including lung cancer. Different preclinical studies have shown the antitumor activity of various medicinal plant extracts against lung cancer. *Melissa oficinalis* L. was reported with a potential antitumoral effect against various cancer cell lines, one of the most representative activities being elicited on the A549 lung cancer cell line. It was demonstrated that the EO (essential oils) at different doses (0, 5, 20, 100, 250, 500, and 1000 µg/mL) possess a high potency to inhibit proliferation of lung cancer cells in a dose-independent manner when compared to a positive control (20 µg/mL Toxol-Paclitaxel (plant-derived chemotherapeutic anti-cancer drug from Taxus brevifolia)), indicating that an optimal dose is more relevant than a maximally tolerated one [67]. Miladi et al. examined the EOs of *Rosmarinus officinalis* L. and *Tymus vulgaris* L. for their in vitro cytotoxic activity against A549 human lung cancer. They revealed that after 48 and 72 h exposure, EO obtained from *Rosmarinus officinalis* L. strongly inhibited the proliferation of A549 cells with an IC_50_ = 8.50 ± 0.01 µg/mL and EO obtained from *Tymus vulgaris* L. with an IC_50_ = 10.50 ± 0.01 µg/mL, respectively [68]. Mohammad-Hossein et al. investigated the properties of the ethanolic extract of *Thymus kotschyanus* L. against A549 lung cancer cell lines. Results indicated that *Thymus kotschyanus* L. extract (10, 5, 2.5, 1.25, 0.63, 0.31, 0.15, and 0.08 mg/mL) inhibited lung cancer cell growth and viability in a dose- and time-dependent manner [69]. A recent study highlights that nargenin nanoemulsion (a flavonoid mainly found in citrus and also grapefruit) presented a high efficiency against A549 human lung cancer cell lines. The nanoemulsion (0.39, 1.56, 6.25, 25, and 100 μg/mL) elicited a concentration-dependent cytotoxicity in A549 lung cancer cells and conducted cell arrest in the G2/M and sub-G1 phases. The authors also concluded that nargenin nanoemulsion increased the caspase-3 and pro-apoptotic Bax protein while decreasing the expression of the anti-apoptotic protein Bcl2 [70]. Poofery et al. tested in vitro three tropical medicinal plants originated from Thailand, *Bridelia ovata Decne* (ethyl acetate extract), *Croton oblongifolius Roxb*. (ethyl acetate extract), and *Erythrophleum succirubrum Gagnep* (ethyl acetate extract and ethanol extract) against A549 human lung cancer cell lines. Cytotoxicity and apoptotic assays showed that all four extracts possessed potential anticancer activity against lung cancer, but the most effective was the ethanolic extract of *E. succirubrum* [71]. Remaining in the field of tropical plants, it was shown that *Ocimum sanctum* L. (originated from Indonesia) elicited an in vitro anticancer effect against A549 cells. The ethanolic extract (50, 70, 100, or 200 ug/mL) significantly decreased the viability of A549 cells. Moreover, at the concentration of 100 μg/mL, it induced the most severe cytotoxicity and apoptosis rate, followed by up-regulation of reactive oxygen species (ROS) and caspase-3 expression and decreased the anti-apoptotic protein Bcl-2 [72]. Yun Tsai et al. indicated that 6-Gingerol (found in *Zingiber officinale* L.) at different concentrations (20–80 μM) suppressed A549 cells’ proliferation in vitro. Moreover, it significantly reduced the tumor size in vivo by administering 0.25 and 0.5 mg/kg/day 6-gingerol to BALB/cNude mice subcutaneously inoculated with A549 tumor cells. Experimental data confirmed that 6-Gingerol increased the expressions of Beclin-1, LC3 I, LC3 II, NCOA4, and TfR1 and down-regulated the expression of USP14, FTH1, GPX4, and ATF4 [73]. Another study demonstrated that carvacrol is a potential candidate for lung cancer therapy. In this regard, the authors tested an oil-in-water carvacrol nanoemulsion (5–100 μg/mL) against doxorubicin-resistant A549 lung carcinoma cells, in vitro as well as in vivo, using an experimental animal model of lung cancer (male nude mice and injected with A549 cells subcutaneously). The in vitro results showed that carvacrol nanoemulsion induced apoptosis and can inhibit proliferation. This was highlighted by an increased level of Bax, caspase 3 and 9 and cytochrome C, cell cycle arrest, decreased CDK2, CDK4, CDK6, Cyclin E, Cyclin D1, and increased p21 protein expression [74].

The group of Yang et al. showed that kukarione, a flavonoid isolated from *Sophora flavescens Aiton*, presented potential antitumor activity against lung cancer both in vitro as well as in vivo. Using different in vitro assays, the authors concluded that kukarione at concentrations of 5, 10, 15, and 20 μg/mL inhibited the proliferation of A549 cells (IC_50_ > 50 μg/mL) and induced apoptosis at the highest concentrations. Moreover, kukarione administering intraperitoneally at doses of 20 and 40 mg/kg/day inhibited the growth of pulmonary cancer (A549 xenograft mouse models athymic BALB/c mice) by up-regulating the pro-apoptotic proteins cleaved-caspase-3, cleaved-caspase-9, and Bax and by down-regulating the antiapoptotic Bcl-2 expression [75].

The literature also reported that some vegetal products from the *Salicaceae* family, same as black poplar buds, presented antitumor activity against different lung cancer cell lines. *Populus alba* L. (also known as white poplar), which has similar properties to *Populus nigra* L., is an example in this direction. Sevgi Gezici et al. described that the EOs obtained from the leaves and flowers of white poplar exhibited a significant antiproliferative effect against A549 (lung adenocarcinoma), MCF7 (breast adenocarcinoma), and H1299 (human non-small cell lung cancer) cell lines with IC_50_ values ranging from 12.05 to 28.16 µg/mL [76]. Pereira et al. demonstrated that *Casearia arborea* L.’s (*Salicaceae* family) essential oils obtained from the fresh leaves by hydro-distillation present a significant cytotoxic effect against the A549 lung cancer cell line. Using the WST-1 assay, it was concluded that concentrations ranging from 0.5 to 20 µg/mL reduce the proliferation of A549 cells (EC50 at 4.0 μg/mL) when compared with the positive control (Doxorubicin 0.01358 μg/mL) [77]. A recent study, conducted by Li Junya et al., showed that Chinese propolis, especially originated from poplar (*Populus* sp.) at concentrations ranging in the interval of 25, 50, and 100 μg/mL significantly inhibited the MDA-MB-231 human breast cancer cell line proliferation, migration, and also invasion [78]. The present study showed that Pg extract produced a dose-dependent decrease of A549 lung adenocarcinoma cells’ viability and migration. Furthermore, at the highest tested concentration, a slight pro-apoptosis effect was noticed. The percentage of early apoptotic cells was increased from 1.34% ± 0.33 (control cells) to 2.68% ± 0.62 (150 µg/mL Pg) after 72 h of incubation with Pg. A cytotoxic effect quantified by the amount of LDH release was also observed following stimulation with Pg extract. At the highest tested concentration (150 µg/mL), a decrease in LDH release was observed, phenomena previously reported for other compounds that induce cell cycle arrest. LDH release can be low at high concentrations tested because the tumor cells do not express proliferative effects [34,79]. Even though LDH release was lower at the highest concentration tested, Pg extract elicited a cytotoxic effect on A549 lung cancer cells, also shown by the images taken following DAPI staining.

## 5. Conclusions

The study concludes that Pg ethanolic extract has a low contribution to trace elements in dietary intake, but the most valuable conclusion is regarding its safety, namely, the tested Pg extract does not produce any harmful effect of metal toxicity during therapeutic application. In the set experimental conditions, Pg extract elicited in a dose-dependent manner antiproliferative and slight pro-apoptotic and cytotoxic potential against the A549 cancer cell line. Further studies need to be conducted that involve an animal model of lung cancer in order to have a full picture of the possible therapeutic potential and benefit in the management of lung cancer.

## Figures and Tables

**Table 1:** Program parameters (T—temperature, t—time, P—power) from the acidic digestion with microwave system MWS-2, realized in three steps.

Program Parameters	**T_1_**	**t_1_**	**P_1_**	**T_2_**	**t_2_**	**P_2_**	**T_3_**	**t_3_**	**P_3_**
	160 °C	15 min	80%	210 °C	15 min	90%	gradual decrease in temperature	15 min	0

**Table 2:** Characteristics for standard calibration curves.

No.	Metal	Wavelength, λ (nm)	Lower Limit, (µg/L)	Upper Limit, (µg/L)	Calibration Curve	R^2^
1	Cu	324.8	3.6	18	y = 0.020731 + 0.016628x	0.9961
2	Pb	283.3	7.4	37	y = 0.001778 + 0.003524x	0.9994
3	As	193.7	13.2	58.1	y = 0.00185 + 0.001544x	0.9927
4	Zn	213.9	1	8	y = 0.071658 + 0.092202x	0.9827
5	Mn	297.5	0.84	4.2	y = 0.007792 + 0.112496x	0.9925
6	Ni	232.0	4.2	34.6	y = 0.033774 + 0.011603x	0.9967
7	Cd	228.8	0.1	2.2	y = 0.004734 + 0.071971x	0.9923
8	Cr	357.9	5	22.0	y = 0.018371 + 0.018435x	0.9961
9	Co	240.7	5.4	29.4	y = 0.008353 + 0.010864x	0.9929
10	Al	309.3	13.2	58.2	y = 0.006978 + 0.00175x	0.9971
11	Fe	248.3	3.6	14.4	y = 0.02274 + 0.013974x	0.9939

**Table 3:** Phenolic compounds in poplar bud samples obtained by LC-MS analysis.

Peak No.	Retention Time R_t_ (min)	[M+H]^+^ (*m*/*z*)	UV λ_max_ (nm)	Compound	Subclass
1	2.96	155	278	Dihydroxybenzoic acid	Hydroxybenzoic acid
2	9.78	155	280	Protocatechuic acid	Hydroxybenzoic acid
3	10.28	355, 163	322	3-Caffeoylquinic acid (Neochlorogenic acid)	Hydroxycinnamic acid
4	11.89	355, 163	322	5-Caffeoylquinic acid (Chlorogenic acid)	Hydroxycinnamic acid
5	12.52	181, 163	320	Caffeic acid	Hydroxycinnamic acid
6	13.52	475, 181	320	Chicoric acid	Hydroxycinnamic acid
7	15.90	447, 271	312, 240	Apigenin-glucuronide	Flavone
8	17.21	477	310, 240	Chrysoeriol -glucuronide	Flavone
9	20.31	389	320, 230	Tremuloidin	Salicin benzoate ester
10	22.17	287	278	Salicin	Hydroxybenzoic acid
11	22.81	271	312, 290	Pinostrobin	Flavanone
12	25.64	529	290, 230	Tremulacin	Salicin ester

**Table 4:** The number of phenolic compounds in the poplar bud sample, expressed in mg/g chlorogenic acid equivalent (CCE).

Peak No.	Rt (min)	Compound	Amount mg/g CEE
1	2.96	Dihydroxybenzoic acid	13.022
2	9.78	Protocatechuic acid	2.674
3	10.28	3-Caffeoylquinic acid (Neochlorogenic acid)	3.382
4	11.89	5-Caffeoylquinic acid (Chlorogenic acid)	8.216
5	12.52	Caffeic acid	4.983
6	13.52	Chicoric acid	30.021
7	15.90	Apigenin-glucuronide	55.828
8	17.21	Chry-glucuronide	48.765
9	20.31	Tremuloidin	30.459
10	22.17	Salicin	8.874
11	22.81	Pinostrobin	18.307
12	25.64	Tremulacin	14.642
Total phenolic content			239.174

**Table 5:** Final concentration of metals (µg/g), as mean of three determinations.

Metal	Co	Cu	Cr	Ni	Fe	Zn	Pb	Mn	Al	As	Cd
Media	*udl	6.66	0.79	3.28	39.00	14.84	*udl	0.59	2109.87	*udl	0.019
SD	-	0.10	0.01	0.01	0.05	0.06	-	0.01	14.02	-	0.001

*udl—under the detection limit.

**Table 6:** IC_50_ values of Pg extract on A549 human lung adenocarcinoma cells at 72 h post-stimulation.

Extract	A549 IC_50_ (µg/mL)
Pg extract	72.49

## Data Availability

All data used to support the findings of this study are included within the article.
